# Geography and elevation as drivers of cloacal microbiome assemblages of a passerine bird distributed across Sulawesi, Indonesia

**DOI:** 10.1186/s42523-022-00219-3

**Published:** 2023-01-16

**Authors:** Rachael L. Joakim, Mohammad Irham, Tri Haryoko, Karen M. C. Rowe, Yohanna Dalimunthe, Syahfitri Anita, Anang S. Achmadi, Jimmy A. McGuire, Susan Perkins, Rauri C. K. Bowie

**Affiliations:** 1 Department of Biology, The City College of New York, 160 Convent Avenue, New York, NY 10031 USA; 2 The Graduate Center of The City University of New York, Biology Program, 365 5Th Ave, New York, NY 10016 USA; 3 Sackler Institute for Comparative Genomics, American Museum of Natural History, New York, NY 10024 USA; 4 The Richard Gilder Graduate School, American Museum of Natural History, New York, NY 10024 USA; 5 Museum Zoologicum Bogoriense, Research Centre for Biology, National Research and Innovation Agency, Jl. Raya Jakarta - Bogor Km 46, Cibinong, 16911 Indonesia; 6 Sciences Department, Museums Victoria, Carlton, VIC Australia; 7 BioSciences Department, University of Melbourne, Parkville, VIC Australia; 8 Museum of Vertebrate Zoology and Department of Integrative Biology, University of California, Berkeley, CA 94720 USA

**Keywords:** Microbiota, Community structure, Elevational shifts, Avian gut microbiome, Sulawesi Babbler, *Pellorneum celebense*, Elevational gradient, Alpha diversity, Beta diversity

## Abstract

**Background:**

Empirical field studies allow us to view how ecological and environmental processes shape the biodiversity of our planet, but collecting samples in situ creates inherent challenges. The majority of empirical vertebrate gut microbiome research compares multiple host species against abiotic and biotic factors, increasing the potential for confounding environmental variables. To minimize these confounding factors, we focus on a single species of passerine bird found throughout the geologically complex island of Sulawesi, Indonesia. We assessed the effects of two environmental factors, geographic Areas of Endemism (AOEs) and elevation, as well as host sex on the gut microbiota assemblages of the Sulawesi Babbler, *Pellorneum celebense,* from three different mountains across the island. Using cloacal swabs, high-throughput-amplicon sequencing, and multiple statistical models, we identified the core microbiome and determined the signal of these three factors on microbial composition.

**Results:**

The five most prevalent bacterial phyla within the gut microbiome of *P. celebense* were *Proteobacteria* (32.6%), *Actinobacteria* (25.2%), *Firmicutes* (22.1%), *Bacteroidetes* (8.7%), and *Plantomycetes* (2.6%). These results are similar to those identified in prior studies of passeriform microbiomes. Overall, microbiota diversity decreased as elevation increased, irrespective of sex or AOE. A single ASV of *Clostridium* was enriched in higher elevation samples, while lower elevation samples were enriched with the genera *Perlucidibaca* (Family *Moraxellaceae*), *Lachnoclostridium* (Family *Lachnospiraceae*), and an unidentified species in the Family *Pseudonocardiaceae*.

**Conclusions:**

While the core microbiota families recovered here are consistent with other passerine studies, the decreases in diversity as elevation increases has only been seen in non-avian hosts. Additionally, the increased abundance of *Clostridium* at high elevations suggests a potential microbial response to lower oxygen levels. This study emphasizes the importance of incorporating multiple statistical models and abiotic factors such as elevation in empirical microbiome research, and is the first to describe an avian gut microbiome from the island of Sulawesi.

**Supplementary Information:**

The online version contains supplementary material available at 10.1186/s42523-022-00219-3.

## Background

The complex relationship between microbial symbionts and their hosts is a functionally important, medically relevant, and often understudied component of global biodiversity. In every habitable natural system, there exists communities of microscopic organisms, including those residing in and on other organisms. Humans and other vertebrates harbor communities of microbes in the gastrointestinal (GI) tract known as the gut microbiota that directly contribute to nutrient uptake, immune function, and fitness plasticity in stochastic environments [1–5]. Studies of vertebrate hosts report various ecological and evolutionary factors driving gut microbiota community assemblage, including diet [6–8], sex [9], reproductive behavior [10], habitat type [11], movement [12] and host phylogeny [13–16]; although many of these factors are interrelated in terms of microbe-specific selection pressures. However, as the majority of the vertebrate gut microbiota literature focuses heavily on mammalian systems [17, 18], there is a significant knowledge gap in terms of how these processes affect other major taxa. Additional studies within non-mammalian hosts, particularly in birds, will allow for generalizable insights regarding host-gut micriobiota dynamics across vertebrates. Avian hosts provide an ideal study system as they are known vectors of human diseases (e.g. West Nile Virus, High-pathogenic Bird Flu), demonstrate diverse ecologies and mating systems, and serve as model systems for comparative analyses in ecology and evolutionary biology [19].

Songbirds (Order: Passeriformes) make up more than 50% of global bird diversity [20]. In general, the gut microbiota of Passeriformes are dominated by the bacterial phyla *Firmicutes*, *Actinobacteria*, *Tenericutes*, *Bacteroidetes*, and *Proteobacteria*, with a particularly high abundance of *Firmicutes* in bird species that feed on insects [21–26]. Comparative field studies have suggested that host phylogeny is the most significant, albeit weakly associated [19], driver influencing the gut microbiome structure of birds. This relationship is independent of other factors such as diet or habitat [6, 19, 21, 26–28]. While placental mammals inoculate their offspring with a portion of their microbiota through live birth, avian young are exposed to microbes in nesting material and receive parental microbiota via incubation and parental saliva [12, 29], suggesting birds are more susceptible to environmental variation in microbial source pools (e.g., due to geographic or ecological gradient effects) than viviparous organisms [15, 19, 26, 28]. While evidence suggests only small differences in avian microbial communities for intraspecific bird populations from both temperate and tropical locations [15, 20, 21, 26, 30], compositional microbiome differences were found between syntopic resident and migratory populations within a species (the barn swallow, *Hirundo rustica* [12]). Whether these differences were due to migration stress or region-specific microbe uptake is difficult to determine. An alternative approach would be to compare the microbial composition within allopatric populations of a single, widespread avian host species, occurring across a repeated environmental gradient (e.g., elevation). To resolve if region-specificity affects the microbial composition of avian hosts in Southeast Asia, we sampled one passerine species in three areas of endemism across a 1000 m elevational range on the island of Sulawesi, Indonesia.

**Fig. 1:**
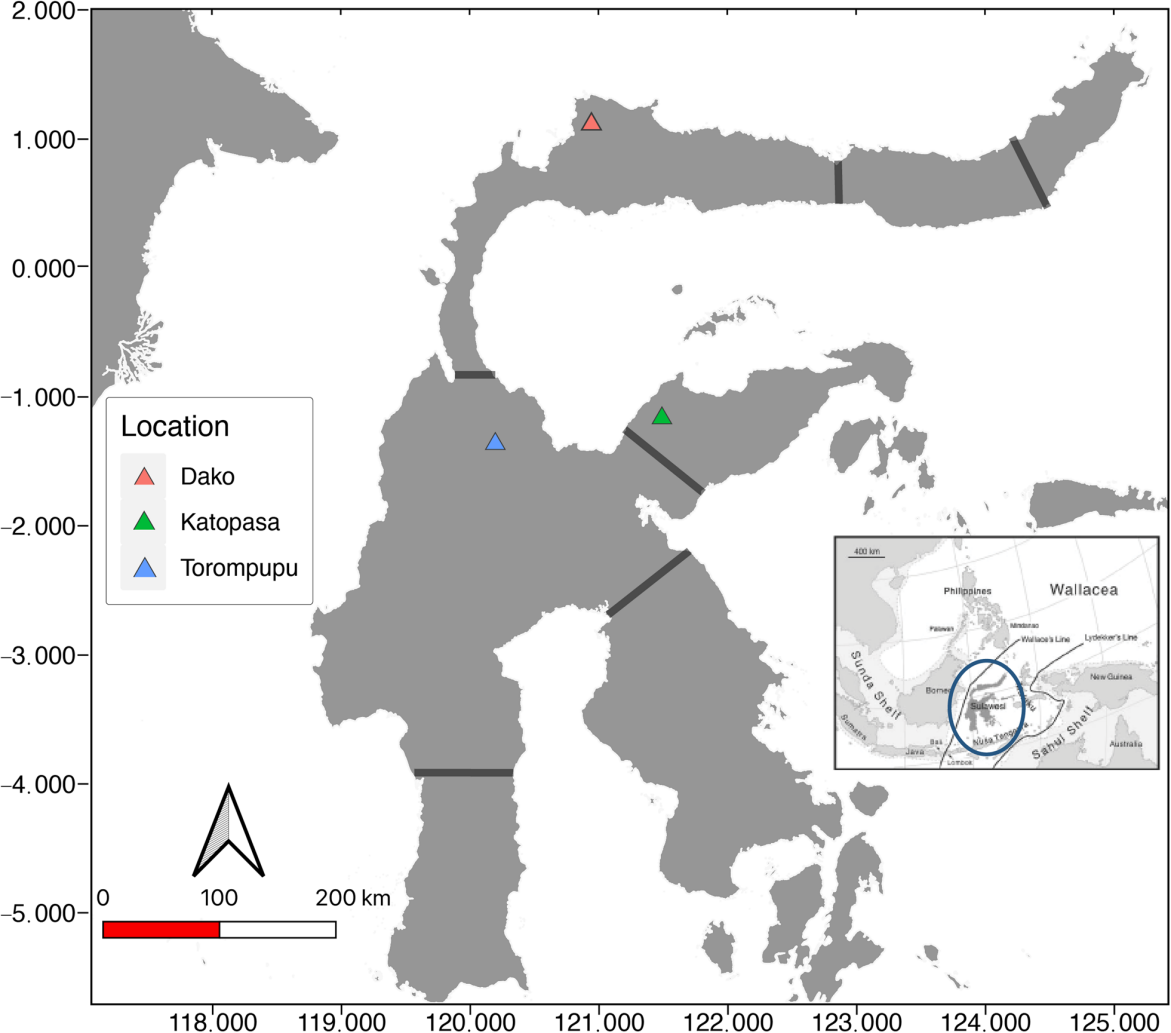
Map of collecting sites on the island of Sulawesi, Indonesia. Grey bars indicate the seven Areas of Endemism (AOEs) described in [32]. The axis values are in degrees

Sulawesi is the eleventh largest island in the world (Fig. 1) and has a complex geological history, with different landmasses accreting and breaking apart over the past 30 million years [31, 32]. Recent studies have revealed Sulawesi remained partially submerged until less than 1 MYA [32]. As a result of these processes, seven different geographically-isolated Areas of Endemism (AOEs) have been identified, supported by clear species boundaries within terrestrial vertebrates [33–35]. Sulawesi is also unusual for an island in that it harbors 25 high-elevation mountains (> 2000 m), which have likely contributed to its unusually high percentage of endemic flora and fauna [31, 33, 36, 37]; endemic species make up approximately 48% of all birds and 36% of all mammals described on the island [38]. The mountains on Sulawesi naturally encompass steep gradients in abiotic variables over short distances, making it possible to rapidly sample multiple individuals of a single species spanning a broad environmental cline. The replicate sampling of high-elevation mountains on Sulawesi provides an opportunity to investigate how avian gut microbiome diversity is shaped by both geography (allopatric isolation on different mountains) and steep clines in abiotic variables (elevational gradients).

Evidence for a relationship between elevation and gut microbial diversity and abundance in mammals, squamates, and birds has been mixed [21, 30, 39–42]. However, we predict a decrease in both diversity and relative abundances of microbial taxa in individuals with increasing elevation, corresponding to the lower diversity and abundance of prey, parasites, and environmental microbiota found at higher elevations [30, 40, 43, 44]. Further, although AOEs on Sulawesi represent distinct population barriers for terrestrial vertebrates, this may not extend to volant organisms and their gut microbiota. We therefore predicted that higher elevation would be negatively correlated with microbial diversity and relative abundances of dominant taxa, and that elevation would be a more significant factor in microbial composition than area of endemism within intraspecific populations of endemic Sulawesi passerines.

To determine the effect of AOE and elevation on avian gut microbiota assemblages within these montane systems, we chose the Sulawesi babbler (*Pellorneum celebense*) as a focal taxon. This species is a common, endemic insectivorous passerine widespread throughout the island, where it occupies dense forest undergrowth from sea-level to 1500 m in elevation, and was the most abundant species found [45–47]. We sampled *P. celebense* individuals along 1000 m elevational transects on three mountains, each within a different AOE, to determine if the diversity and relative abundances of gut microbial assemblages correlate with both elevation and area of endemism on the island of Sulawesi (Fig. 1). Host sex was also included in analyses to account for potential sex-dependent variation*.* We tested the hypotheses that *H*_*1*_*: diversity and abundance of* P. celebense *gut microbiota is negatively correlated with elevation; and H*_*2*_*: areas of endemism do not have significant effect on host intraspecific microbial variation.*

## Results

A total of 4339 ASVs from 40 *Pellorneum celebense* individuals were recovered for downstream analyses. The R package “decontam” did not find any reads from positive or negative controls that were found in all corresponding samples, so no matching reads were removed from the dataset. After transforming raw read counts and removing low abundance ASVs, 3445 were retained in the transformed dataset, with 3028 ASVs recovered after removing individuals with < 3000 reads (n = 2) and rarefying the remaining 38 individuals to the lowest read count (n = 3389 reads, Table 1). A t-test comparing ASV richness by sample between the transformed and rarefied datasets did not reveal a difference in microbial richness (t = 0.81, *p* = 0.42), so only the results from unrarefied data are reported.

**Table 1:** Comparison between amplicon sequence variant (ASV) preprocessing of the original 4339

	ASVs	Samples	Mean ASV per sample
Transformed	3445	40	95.3
Rarified	3028	38	86.1

Transformed reads were log transformed with all samples below 0.00001 removed, while rarified reads were randomly subset to 3028 reads per sample

### P. celebense microbiome composition

**Fig. 2:**
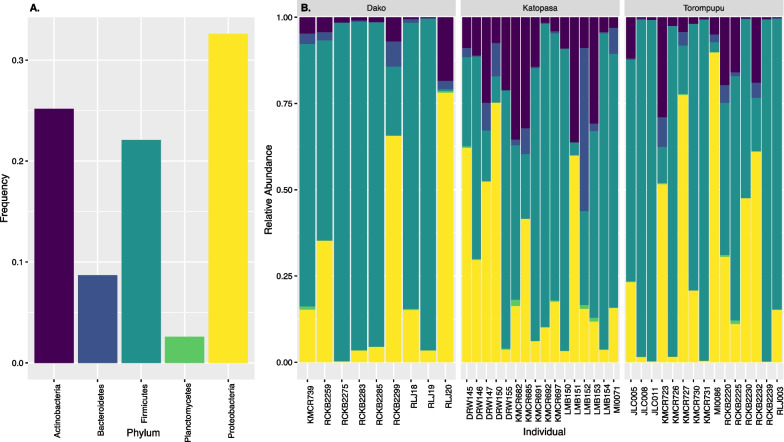
Absolute frequencies in all samples (**A**) and the relative abundances of each individual (**B**) of the 5 most abundant phyla of ASVs from the *P. celebense* cloacal microbiome compositional dataset, identified using the 16S SILVA ribosomal RNA gene database

Only one ASV, an unidentified member of the *Enterobacteriaceae* family (Phylum *Proteobacteria*), was found in 90% of individuals. An additional ASV in the *Enterococcaceae* family (Phylum *Firmicutes*) was shared among 90% of the Torompupu individuals. The five most prevalent bacterial phyla within the gut microbiome of *P. celebense* were *Proteobacteria* (32.6%), *Actinobacteria* (25.2%), *Firmicutes* (22.1%), *Bacteroidetes* (8.7%), and *Plantomycetes* (2.6%). The relative abundances of these phyla by individual are visualized in Fig. 2.

### Trends in P. celebense microbe diversity

**Fig. 3:**
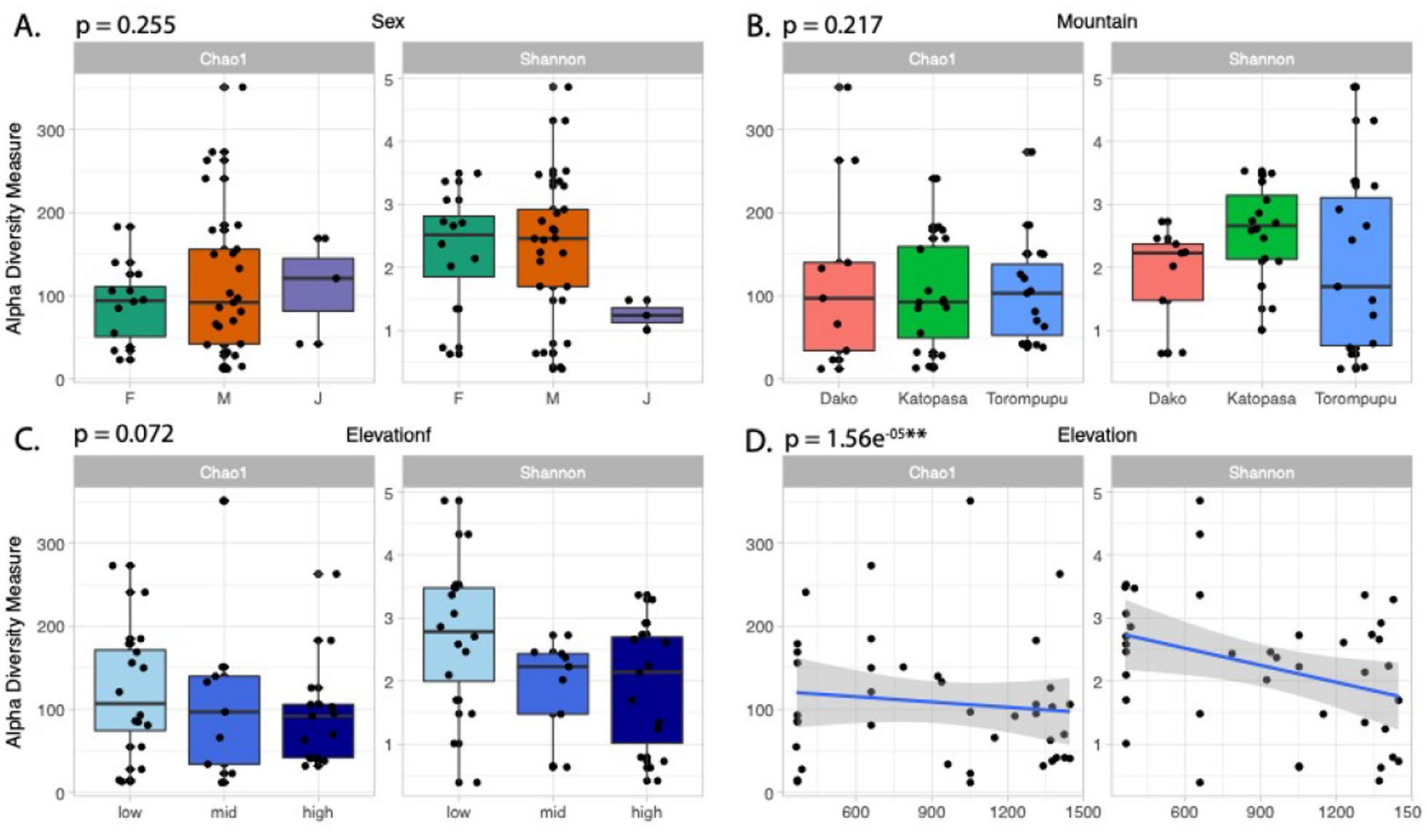
Microbial alpha diversity plots and associated *p* values using number of ASVs and Shannon Index compared by the variables: **A** sex (adults) and juveniles, **B** mountain, **C** elevational as a factor (low =  < 700 m, mid = 700–1200 m, high =  > 1200 m), and **D** continuous elevation

**Fig. 4:**
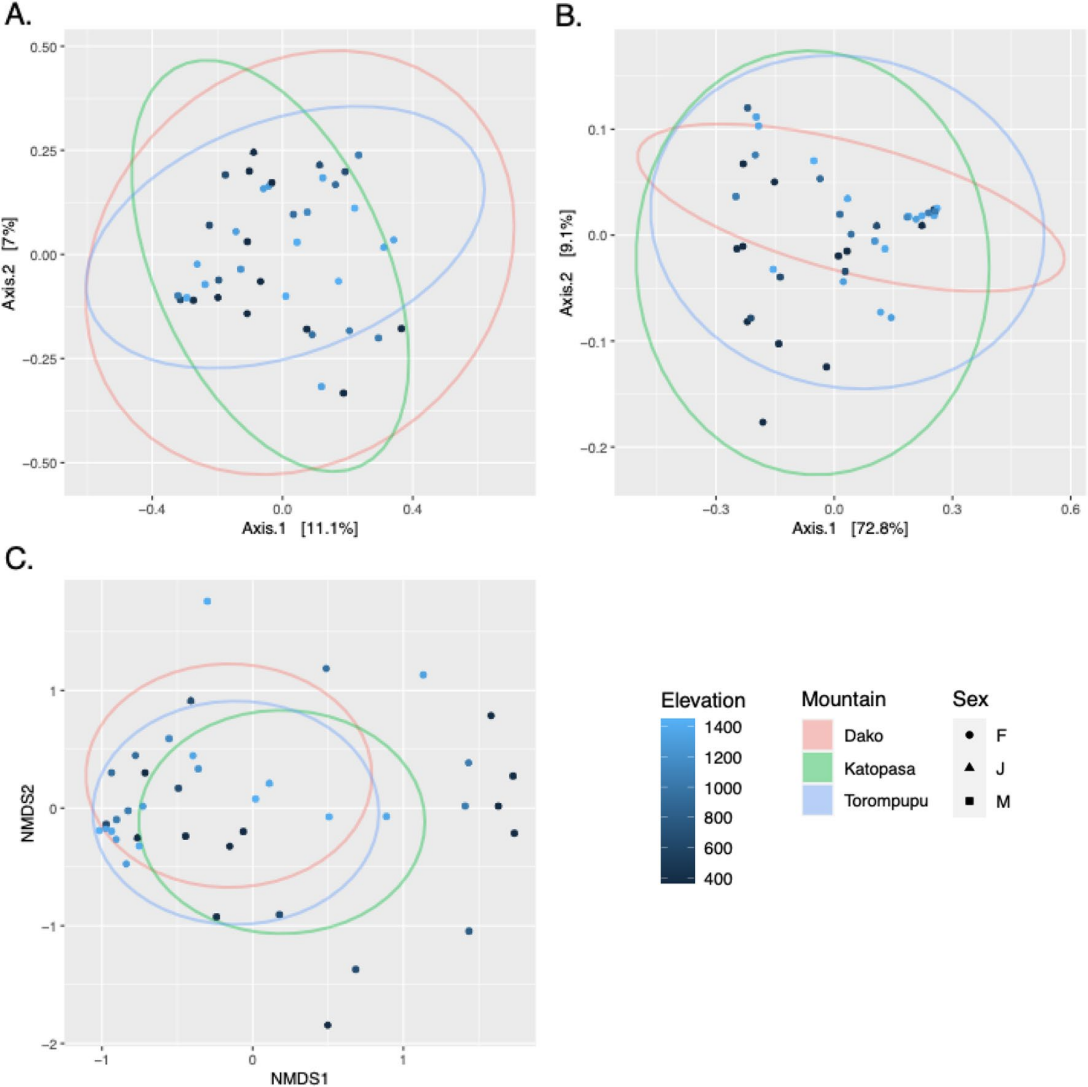
Beta-diversity PCoA plots using unweighted Unifrac (**A**), weighted Unifrac (**B**), and NDMS (**C**) ordinations. Stress value for the NDMS was 0.190. Individuals are represented by a point shaped by sex and colored by elevation. Variations by mountain are represented by ordination ellipses

*By Sex and Mountain* To determine if host sex or AOE is directly correlated with changes in alpha diversity, ANOVAs of individual Shannon indices using mountains and sex as explanatory variables were run. Results did not reveal differences in mean group diversity (F_Mountain_ = 1.594, p_Mountain_ = 0.217; F_Sex_ = 1.420, p_Sex_ = 0.255, Fig. 3). These results did not change when these ANOVAs were replicated using Chao1 as the diversity index (Fig. 3). However, PERMANOVAs comparing Unifrac beta diversity indices indicated a slight difference in microbial membership (unweighted Unifrac) among mountains (F = 1.216, r^2^ = 0.062, *p* = 0.062, Fig. 4a), though this difference was no longer significant when relative abundance of ASVs (weighted Unifrac) was included in the analysis (F_w_ = 1.283, r^2^_w_ = 0.065, *p*_w_ = 0.254, Fig. 4b). A PCoA plot of weighted Unifrac distances revealed a higher clustering of samples from Dako, suggesting that microbial communities of this sample population may have more phylogenetic similarity than populations on the other mountains, as a non-dimensional ordination did not show the same clustering of Dako samples (Fig. 4c).

**Fig. 5:**
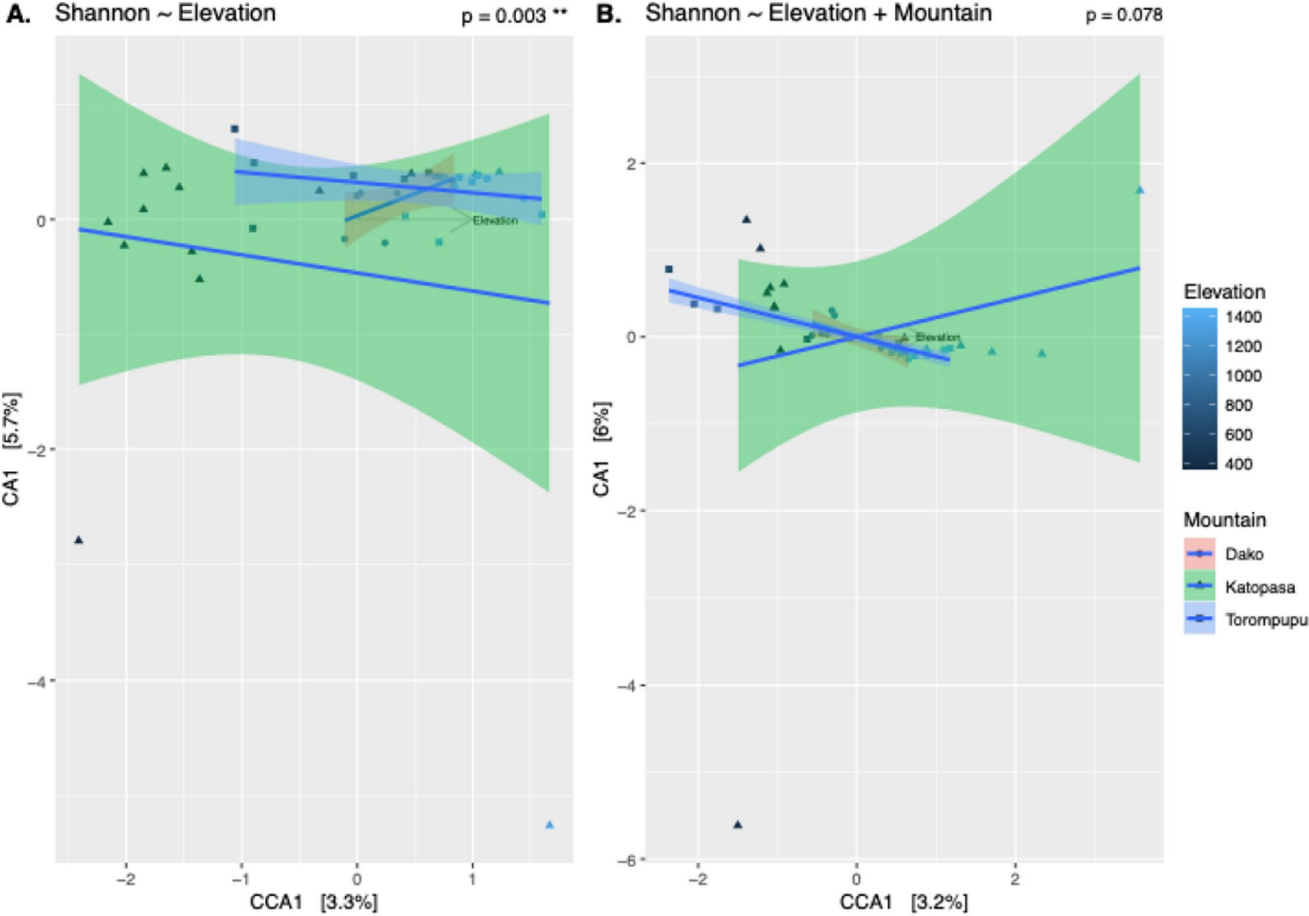
Canonical Correspondence Analyses (CCAs) using only elevation as an environmental gradient (**A**) and with both elevation as a gradient and mountain as a conditional variable (**B**). Mountains are represented as separate regression lines, with shaded areas represent 95% confidence intervals. *P* values of each model indicates that the correlation between microbial diversity and elevation is less significant when sampling location is considered

*By Elevation* While an ANOVA of individual Shannon diversity by elevational category did not show a significant decrease in mean diversity as elevation increases (F = 5.174, *p* = 0.072, Fig. 3c), a linear regression using numerical elevation data revealed a significant decrease as elevation increases (t = 4.967, *p* = 1.56e^−05^, Fig. 3d). PERMANOVAs of beta diversity indices revealed a significant difference by elevational category only in microbial composition (weighted Unifrac), not membership alone (unweighted Unifrac) (F_w_ = 2.521, r^2^_w_ = 0.120, *p*_w_ = 0.044; F = 1.180, r^2^ = 0.060, *p* = 0.071). A PERMANOVA of CCA ordination residuals testing the effect of elevation as an environmental gradient also revealed a clear negative trend between elevation and microbial relative abundance (F = 1.230, *p *= 0.004, Fig. 5a). When this analysis was repeated adding mountain as a conditional variable, these changes in abundance became less significant, though plotting regression lines suggests a continued negative correlation with elevation (F_m_ = 1.202, *p*_m_ = 0.077, Fig. 5b).

### Modeling interacting factors

To determine if mountain or host sex is producing a random effect potentially confounding the influence of elevation on microbial assemblages, three robust mixed-effect models were run with elevation as the predictor effect and mountain and sex as separate and interacting random effects. Because robust models do not generate *p* values, the significance of models are confirmed if the slope and 95% CI intervals do not intercept 0 [48]. All three models were significant under these conditions and resulting predictor plots revealed identical negative correlations between microbial diversity and elevation, regardless of which categorical variable was used as the random effect (Additional file 1: Fig. S2 and Table S2).

### Elevational effects on microbe taxonomic abundance

**Fig. 6:**
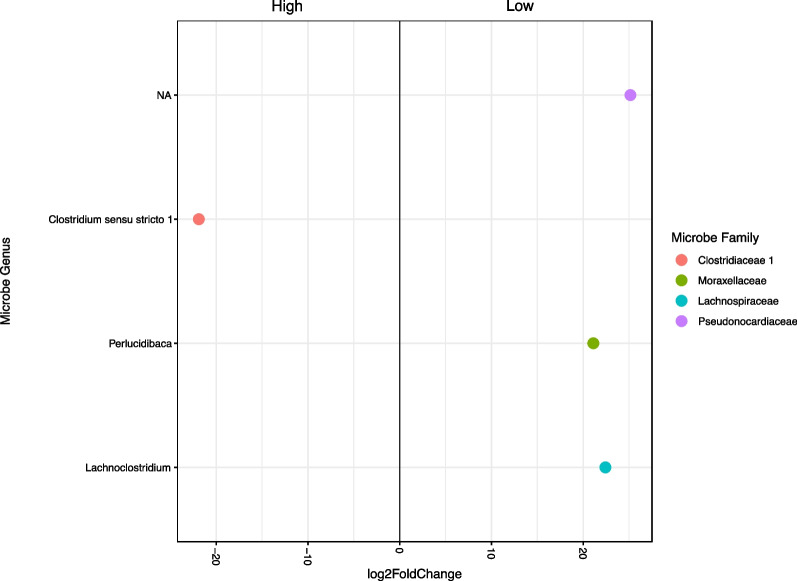
Differential abundances between low and high elevational categories. ASV’s enriched in higher elevations fall to the left side of the x-axis, while those enriched in lower elevations fall to the right

To determine which microbial families changed in abundance at each end of the elevational gradient, logfold values were only compared between the “low” and “high” elevation categories. The families *Pseudonocardiaceae, Lachnospiraceae*, and *Moraxellaceae* were significantly more abundant at lower elevations, whereas *Clostridiaceae* was enriched at higher elevations (Fig. 6).

## Discussion

In this study, we determined the effects of elevation, mountain (representing independent AOEs), and sex on microbial assemblages in a Sulawesi songbird, *Pellorneum celebense*. In general, cloacal microbiota appeared to decrease in abundance and diversity as elevation increases, regardless of mountain or host sex. Surprisingly, there seems to be no influence of host sex on microbiome composition in this system. While a PERMANOVA comparing microbial membership (unweighted Unifrac distances) did reveal a significant influence of mountain, the most likely explanation is that this species was only found above 900 m on Mt. Dako. Therefore, the differences are most likely attributed to a lack of sampling at lower elevations at this locality, as this significance was lost when abundance-weighted microbial composition (weighted Unifrac distances) was compared (Additional file 1: Fig. S1).

While previous avian gut microbiota studies did not reveal correlations with elevation, decreases in microbial alpha and beta diversities at higher elevations are also seen across populations of the toad-headed lizard, *Phrynocephalus vlangalii* [39]. This decrease is thought to be influenced by hypoxic conditions resulting from decreased oxygen partial pressure, though it is worth noting the elevational range of *P. vlangalii* (2900–4250 m) is much higher than that of *P. celebense*. Conversely, a study of high-altitude pikas (*Ochotona curzoniae*) found higher diversity and functional enrichment as elevation increased [40], while studies of Tibetan ruminants (*Bos spp.* and *Ovis spp.*) and mesquite lizards (*Sceloporus grammicus*) did not find any relationship between diversity and elevation that was not consistent with subsequent dietary shifts [49, 50]. While we can assume the decrease in diversity at higher elevations is associated with lower diversity of invertebrate prey species, a lack of dietary analyses prevents a definitive conclusion. Additionally, selection pressures, especially those related to oxygen levels and air pressure, are likely increasing as elevation increases. Expanding elevational analyses to include avian host species with a wide elevational range, especially those differing in feeding guilds, would further validate this negative correlation with microbial diversity at the community scale.

Observed patterns from specific microbial taxa found in *P. celebense* cloacal samples also offer insights into the underlying factors influencing the microbiome community. *Clostridium *sensu stricto* 1* is an obligate anaerobic fermenter metabolizing a range of compounds such as carbohydrates, amino acids, alcohols, and purines [51]. The increased presence of *Clostridium* in higher elevation hosts may suggest an increased dependency on microbial symbionts for metabolism due to lower oxygen levels, as microbes related to host metabolic pathways were also seen in higher proportions in high-elevation *S. grammicus* lizard populations [50]. The genus *Perlucidibaca* has only been isolated from aquatic samples, which does not provide a clear biological explanation for its increased abundance in hosts at low elevations [52]. A possible explanation is that these microbes are present in sampling sites near bodies of standing water (which are typically seen only in lower elevations), though no environmental samples were collected in this study so this could not be confirmed. Members of the family *Pseudonocardiaceae* are known to produce antibacterial metabolites, especially in high nitrogen environments [53, 54]. The genus *Lachnoclostridium* is associated with metabolism of similar metabolites as *Clostridium*, but has been identified as an indicator of early stages of colorectal cancer and therefore may indicate localized immune activity in the cloaca [55, 56]. Both families were also significantly more abundant in hosts at lower elevation, which may suggest increased immune activity. Because the majority of functional analyses on gut microbes are human-based, however, these potential associations are merely speculative. Future gut microbiota studies incorporating measured immune activity could discern if these microbes are associated with avian immune function.

## Conclusions

The results of this study illuminate the importance of assessing abiotic and biotic factors in empirical gut microbiota research by documenting intraspecific variation seen in wild host populations. Studies focusing on host phylogeny and diet in wild systems may miss the potential confounding effects of environmental factors if they are not included in these analyses. In the absence of elevational data, this dataset would reveal limited spatial or sex-dependent variation in microbial communities. Sulawesi montane ecosystems provide an ideal study system of an isolated, endemic avian community. By incorporating an elevational gradient and testing for interacting factors, we show that the cloacal microbiota membership, structure, and overall abundance in *P. celebense* populations significantly decreases in higher elevations in all three different areas of endemism that were sampled. Additionally, the higher abundance of metabolic microbe ASVs at high elevations suggests altitude-related shifts in community structure. Future community studies can confirm if these elevational shifts are consistent across host feeding guilds and phylogeny. This study lays the foundation for future work on montane host communities on Sulawesi, and contributes to a growing global microbiome dataset by providing the first report of avian gut microbiota from this remarkably unique island.

## Methods

### Study region

Each of the three mountains surveyed shared general habitat gradients relative to elevation (Fig. 1). We categorized elevations under 1000 m as lowland forest, dominated by large canopy trees with thick undergrowth. Above 1000 m we observed mossy transitional forests, with understory dominated by rattan vines (subfamily *Calamoideae*). Summit ecosystems (> 1600 m) are categorized as mossy forests with a sparse understory of *Rhododendron spp.*, though no *P. celebense* individuals were found in ecosystems above 1450 m.

- *Gunung Torompupu* NW central Core AOE. Summit is 2495 m. Located west of the Palu-Koro fault, which has been shown to be a species boundary for terrestrial vertebrates [34]. The understory at all elevations was dominated by dense rattan.
- *Gunung Katopasa* Eastern peninsula AOE. Summit is 2835 m. Sampling sites between 700 and 1300 m included fire damage and clearings for small plantations.
- *Gunung Dako* Northern Peninsula AOE. Summit is 2260 m. Because the mountain is more accessible than most, lower elevation habitat was fragmented by small plantations with cleared understories. Given that this species specializes in dense forest undergrowth, the level of cultivation likely explains why we did not observe *P. celebense* below 900 m on this mountain.

### Sample collection

Birds were sampled during three, month-long expeditions during the dry seasons between 2017 and 2018 (Fig. 1, Table 2) using mist-net transects spanning elevational gradients at each mountain. Once captured in mist-nets, individuals were promptly removed and placed in cloth holding bags while transported to camp for processing. To profile the avian gut microbiota, cloacal swabs (Copan Diagnostics, Murrieta, CA) were collected from live animals once at camp. First, 3% H_2_O_2_ and a fresh KimWipe^®^ was used to sterilize the skin surrounding the cloaca. A sterile flocked nylon swab was gently inserted, turned ½ rotation clockwise and ½ rotation counterclockwise, and placed into a tube of 96% ethanol. This reagent was chosen over RNAlater^®^ as it is less likely to degrade DNA when stored at warmer temperatures, which is unavoidable during remote tropical fieldwork [57, 58]. A tube of ethanol was used as a negative for every new site to account for potential reagent contamination. Individuals were sexed by dissection and visual inspection of gonads. If there were no developed ova or testes and the skull was not fully ossified, birds were defined as juvenile. Upon returning from the field, cloacal swabs were stored in a – 80° freezer until processed. All sampling protocols were approved by institutional animal care and use committees at the University of California, Berkeley (AUP-2016-04-8665-1) and the American Museum of Natural History (AMNHIACUC-20171020). Research permits (Surat Izin Penelitian) for three expeditions undertaken in 2017 and 2018 were obtained from Ministry of Research, Technology and Higher Education (KEMENRISTEKDIKTI) (no. 213/SIP/FRP/E5/Dit.KI/VIII/2017), with sample export documents for each expedition provided by the Research Center for Biology, Indonesian Institute of Sciences (LIPI).

**Table 2:** Sampling site information for *Pellornuem celebense* cloacal swabs used in this study

Mountain	Dako	Katopasa	Torompupu
Total n	9	15	16
Summit Elevation (m)	2260	2835	2495
Area of Endemism (AOE)	N Peninsula	E Peninsula	NW Central Core
Sampling range (m)	924–1406	364–1340	660–1446
Sampling date	Jul-18	Aug-17	Nov-17

### Microbiome sequencing

DNA extractions were conducted using the MoBio^®^ Power Soil kit (MoBio Laboratories Inc., Carlsbad, CA) with an extra wash step to maximize DNA recovery. DNA was PCR-amplified in triplicate using the Earth Microbiome Project’s 16S rRNA PCR protocol primers 515RB (5′-GTGYCAGCMGCCGCGGTAA-3′) and 806RB (5′-GGACTACNVGGGTWTCTAAT-3′) and sequenced on the Illumina^®^ MiSeq platform, generating 250 base pair paired-end amplicon reads [59, 60]. PCRs and sequencing, including of negative controls, were performed at the Argonne National Laboratory Sample Processing Facility (Lemont, IL, USA).

### Data preprocessing

Preprocessing of raw reads was conducted using packages implemented in QIIME2 v.2018.11 [60]. With the DADA2 plugin, single-end reads were trimmed and chimeric sequences were removed before being paired and classified as Amplicon Sequence Variants (ASVs) [61]. A multiple sequence alignment of paired-end reads was generated using the ‘mafft’ program [62], and FastTree was used to create a midpoint-rooted phylogenetic tree [63]. Taxonomic identification of each ASV was generated using the Naïve Bayesian q-2 feature classifier trained on the 16S SILVA 132 ribosomal RNA gene database [64]. Downstream analyses were conducted in RStudio v.1.2.1335. The R package ‘decontam’ was used to assess potential contamination using field and laboratory controls [65]. Reads that matched chloroplast and host mitochondrial sequences were then removed. Due to the compositional nature of Illumina MiSeq data, [66] reads were transformed to relative abundances and ASVs with a relative abundance of < 0.00001 were removed for certain downstream analyses using the R package ‘phyloseq’ [67]. However, a rarefied dataset using the ‘phyloseq’ command rarefy_even_depth was analyzed in parallel to rule out potential effects of these preprocessing steps.

### Statistical analyses

All statistical analyses and visualizations were performed in R 4.1.2 (R Core Team 2020). The top 20 microbial phyla were identified, and the shared microbial taxa of all samples were determined using the core_members function of ‘phyloseq’. Mountain and host sex were used as categorical variables, while elevation was analyzed as either a continuous or categorical variable: “low” for samples from elevations under 700 m (n = 16), “mid” for 700–1200 m (n = 9), and “high” for elevations over 1200 m (n = 15). Alpha diversity means were calculated using Shannon diversity and Chao1 diversity indices using the estimate_richness function of ‘phyloseq’ and compared using ANOVAs (for categorical factors) with post-hoc Tukey tests and a general linear model (for elevation). Beta diversity indices were calculated as the sum of phylogenetic branch lengths (Unifrac distances) to compare variances in microbial membership (unweighted Unifrac) and composition (abundance-weighted Unifrac) among categorical factors using PERMANOVAS with the adonis function [68]. We also included an NDMS ordination based on Bray–Curtis distances. To evaluate elevation as a continuous environmental gradient, Canonical Correspondence Analyses (CCAs) were run [69], using categorical factors as conditional variables. To determine directional correlations between microbial diversity and elevation, robust mixed-effect models were created and evaluated using the R package “robustlmm,” as the robust model is more appropriate than other mixed models for datasets with small sample sizes and high degrees of freedom [48]. We used Shannon diversity index as the response variable in this model to detect shifts in ASV abundance and richness, using elevation as the continuous fixed effect and combinations of sex and mountain as random effects. DESeq2, a logistical regression of dispersions weighted by normalized mean counts of unfiltered taxonomic data [70], was used to assess *which* microbial taxa changed in abundance among mountain, host sex, and elevational category.

## Supplementary Information

**Additional file 1: Table S1. **Specimen data for samples used in this study. **Fig. S1. **Histogram of total elevation values for each specimen, and total values by mountain. The inconsistency in elevational sampling gradient at each mountain explains variation seen in mountain-based comparisons. **Fig. S2.** PCoAs of Unweighted and Weighted unifrac distances by mountain. **Fig. S3. **Robust linear model residual effect plot with elevation as the predictor effect. Tic marks along the x-axis represent elevations of individual data points. All plots were identical, regardless of whether mountain or host sex were set as conditional variables. **Table S2. **Slope and confidence intervals for robust mixed-effect models using elevation as an environmental gradient and mountain and sex as random effects. All three models were not significant as the confidence intervals do not intersect zero [48].

## Data Availability

All data files and reproduceable code can be found at https://github.com/rjoakim/babbler_microbiome
