# Supplementary material for: Geography and elevation as drivers of cloacal microbiome assemblages of a passerine bird distributed across Sulawesi, Indonesia
Source: Anim Microbiome. 2023 Jan 16;5:4. doi: 10.1186/s42523-022-00219-3 (PMC9841722; doi:10.1186/s42523-022-00219-3)
Supplement: Supplementary file 1 — Additional file 1: Table S1. Specimen data for samples used in this study. Fig. S1. Histogram of total elevation values for each specimen, and total values by mountain. The inconsistency in elevational sampling gradient at each mountain explains variation seen in mountain-based comparisons. Fig. S2. PCoAs of Unweighted and Weighted unifrac distances by mountain. Fig. S3. Robust linear model residual effect plot with elevation as the predictor effect. Tic marks along the x-axis represent elevations of individual data points. All plots were identical, regardless of whether mountain or host sex were set as conditional variables. Table S2. Slope and confidence intervals for robust mixed-effect models using elevation as an environmental gradient and mountain and sex as random effects. All three models were not significant as the confidence intervals do not intersect zero [48]. [file 42523_2022_219_MOESM1_ESM.docx]

Supplementary:

Table S1: Specimen data for samples used in this study.

| **SampleID** | **Mountain** | **Sex** | **Elevation (m)** | **Date** |
| --- | --- | --- | --- | --- |
| **DRW145** | Katopasa | F | 368 | Aug-17 |
| **DRW146** | Katopasa | M | 368 | Aug-17 |
| **DRW147** | Katopasa | M | 386 | Aug-17 |
| **DRW150** | Katopasa | M | 368 | Aug-17 |
| **DRW155** | Katopasa | F | 1312 | Aug-17 |
| **JLC005** | Torompupu | M | 660 | Nov-17 |
| **JLC008** | Torompupu | J | 660 | Nov-17 |
| **JLC011** | Torompupu | M | 660 | Nov-17 |
| **KMCR682** | Katopasa | M | 401 | Aug-17 |
| **KMCR685** | Katopasa | F | 364 | Aug-17 |
| **KMCR691** | Katopasa | F | 1312 | Aug-17 |
| **KMCR692** | Katopasa | F | 1312 | Aug-17 |
| **KMCR697** | Katopasa | M | 1228 | Aug-17 |
| **KMCR723** | Torompupu | M | 660 | Nov-17 |
| **KMCR726** | Torompupu | M | 1369 | Nov-17 |
| **KMCR727** | Torompupu | M | 1376 | Nov-17 |
| **KMCR730** | Torompupu | M | 1446 | Nov-17 |
| **KMCR731** | Torompupu | F | 1446 | Nov-17 |
| **KMCR739** | Dako | M | 1406 | Jul-18 |
| **LMB150** | Katopasa | M | 368 | Aug-17 |
| **LMB151** | Katopasa | M | 368 | Aug-17 |
| **LMB152** | Katopasa | F | 368 | Aug-17 |
| **LMB153** | Katopasa | M | 368 | Aug-17 |
| **LMB154** | Katopasa | J | 368 | Aug-17 |
| **MI0071** | Katopasa | M | 1340 | Aug-17 |
| **MI0086** | Torompupu | M | 787 | Nov-17 |
| **RCKB2220** | Torompupu | M | 660 | Nov-17 |
| **RCKB2225** | Torompupu | F | 1369 | Nov-17 |
| **RCKB2230** | Torompupu | J | 1393 | Nov-17 |
| **RCKB2232** | Torompupu | M | 1425 | Nov-17 |
| **RCKB2239** | Torompupu | M | 1423 | Nov-17 |
| **RCKB2259** | Dako | F | 963 | Jul-18 |
| **RCKB2275** | Dako | M | 1052 | Jul-18 |
| **RCKB2283** | Dako | M | 1148 | Jul-18 |
| **RCKB2285** | Dako | F | 924 | Jul-18 |
| **RCKB2299** | Dako | F | 1052 | Jul-18 |
| **RLJ003** | Torompupu | F | 1376 | Nov-17 |
| **RLJ18** | Dako | M | 939 | Jul-18 |
| **RLJ19** | Dako | M | 1052 | Jul-18 |
| **RLJ20** | Dako | M | 1052 | Jul-18 |

Figure S1: Histogram of total elevation values for each specimen, and total values by mountain. The inconsistency in elevational sampling gradient at each mountain explains variation seen in mountain-based comparisons.

Figure S2: PCoAs of Unweighted and Weighted unifrac distances by mountain.


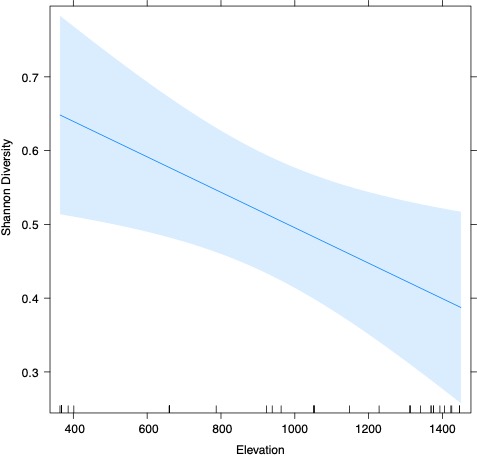


Figure S3. Robust linear model residual effect plot with elevation as the predictor effect. Tic marks along the x-axis represent elevations of individual data points. All plots were identical, regardless of whether mountain or host sex were set as conditional variables.

Table S2: Slope and confidence intervals for robust mixed-effect models using elevation as an environmental gradient and mountain and sex as random effects. All three models were not significant as the confidence intervals do not intersect zero [51].

|  | | **CI intervals** | |
| --- | --- | --- | --- |
| **Model Function** | **Slope** | **Lower** | **Upper** |
| (Shannon ~ Elevation + (1\|Sex)) | -9.0979e­^-4^ | -0.0018 | -6.4870e^-5^ |
| (Shannon ~ Elevation + (1\|Mountain)) | -9.0979e­^-4^ | -0.0018 | -6.4870e^-5^ |
| (Shannon ~ Elevation + (1\|Sex) + (1\|Mountain)) | -9.0979e­^-4^ | -0.0018 | -6.4870e^-5^ |
